# Molecular and Epidemiologic Analysis of Reemergent *Salmonella enterica* Serovar Napoli, Italy, 2011–2015

**DOI:** 10.3201/eid2403.171178

**Published:** 2018-03

**Authors:** Michela Sabbatucci, Anna Maria Dionisi, Patrizio Pezzotti, Claudia Lucarelli, Lisa Barco, Marzia Mancin, Ida Luzzi

**Affiliations:** European Centre for Disease Prevention and Control, Stockholm, Sweden (M. Sabbatucci);; Istituto Superiore di Sanità, Rome, Italy (M. Sabbatucci, A.M. Dionisi, P. Pezzotti, C. Lucarelli, I. Luzzi);; Istituto Zooprofilattico Sperimentale delle Venezie, Padua, Italy (L. Barco, M. Mancin)

**Keywords:** Salmonella enterica serovar Napoli, bacteria, reemergence, environment, food contamination, food safety, vegetables, surface water, waterborne disease, zoonoses, salmonellosis, infections, human infections, molecular analysis, epidemiology, Italy

## Abstract

Human infections with *Salmonella enterica* serovar Napoli are uncommon in Europe. However, these infections represented 5.9% of salmonellosis cases in Italy during 2014–2015. The source of infection is unknown. We analyzed surveillance data and compared strain genetic similarities and found that contaminated vegetables and surface water are probable sources of human infection.

*Salmonella enterica* serovar Napoli has attracted interest since 1982, when chocolate contaminated with this serovar and imported from Italy caused 2 foodborne outbreaks (*1**,**2*). During 2000–2006, a 140% increase in *Salmonella* Napoli cases was reported in Europe (*3*), most (87%) in France, Italy and Switzerland. Since 2004, the Rapid Alert System for Food and Feed (https://webgate.ec.europa.eu/rasff-window/portal/) has received 8 reports of *Salmonella* Napoli infection, which involved 6 countries and vegetables from Italy as the source of infection.

In Italy, sporadic cases of infection with *Salmonella* Napoli were reported before and after the first outbreak was reported in 1984 (*4*). The number of cases increased by 28% during 2000–2011 (*5*). Recently, *Salmonella* Napoli has caused waterborne (*6*) and foodborne (*7*) outbreaks. Although studies in other countries (*3*) and Italy (*8**–**10*) investigated the mode of infection transmission, the zoonotic reservoir is still unknown. 

To update epidemiologic trends for *Salmonella* Napoli in Italy, we identified the population at greatest risk for infection and defined putative vehicles of infection, which could assist possible control measures. We also analyzed 2011–2015 surveillance data for human, animal, environmental, food, and feed *Salmonella* Napoli isolates; evaluated associated factors; and compared genetic profiles.

## The Study

We extracted data on February 16, 2017, from the passive, voluntary, laboratory-based, surveillance system Enter-Net (http://www.iss.it/ente) for *Salmonella* spp. isolates from 2011–2015; patient identity was anonymous. Enter-Net is coordinated by the Istituto Superiore di Sanità (Rome, Italy) and contains information on strains from human and environmental samples. We excluded samples from nonhuman and nonenvironmental sources (n = 43), unspecified sample types (n = 21), and undefined *Salmonella* serovars (n = 1,066) from analysis. We obtained data (aggregated for 2011–2012 and 2013–2014) for *Salmonella* spp. isolated from food, animals, and feed by using the passive and voluntary veterinary surveillance system Enter-Vet (http://www.izsvenezie.it/temi/malattie-patogeni/salmonella/enter-vet/).

We evaluated potential epidemiologic risks associated with human and environmental *Salmonella* Napoli isolates and the 5 most frequent nontyphoidal *Salmonella* (NTS) serovars other than Napoli (monophasic variant of Typhimurium, Typhimurium, Derby, Enteritidis, and Infantis) by using simple 2-way frequency tables. Univariate analysis (p<0.05 by χ^2^ test) was based on comparing categories for each variable and excluding missing values. We developed a multiple logistic regression model (Stata version 12; StataCorp LLC, College Station, TX, USA) to evaluate potential confounding among variables. We also used data for patients hospitalized with *Salmonella* Napoli infections or bacteremia as outcomes adjusted for other factors. The top 5 other NTS represented 73% of human *Salmonella* serovars isolated during 2011–2015. Results were consistent when we compared *Salmonella* Napoli with all reported NTS.

We serotyped *Salmonella* spp. isolates by using the White–Kauffmann–Le Minor scheme (*11*) and evaluated genetic relatedness among *Salmonella* Napoli isolates by using pulsed-field gel electrophoresis according to the PulseNet protocol (https://www.cdc.gov/pulsenet/pathogens/protocols.html). We performed dendrogram and cluster analyses by using BioNumerics 7.5 (Applied Maths, Sint-Martens-Latem, Belgium). We scored similarity between chromosomal fingerprints by using the Dice coefficient and used the unweighted pair-group method with arithmetic means, 1.00% tolerance limit, 1.00% optimization, and coefficient of similarity >80% to define clonal relationships between strains.

The monophasic variant of Typhimurium (37.3%), Typhimurium (21.6%), Enteritidis (9.3%), Napoli (4.7%), Derby (3.1%), and Infantis (1.9%) were the most frequent of 331 *Salmonella* serovars identified for 21,486 human records. The proportion of *Salmonella* Napoli cases increased from 4.3% in 2011 to 5.8% in 2015 (p<0.001). *Salmonella* Napoli infection was more common in men, infants, children, and the elderly in northern and southern Italy during July–September than other reported serovars (Table 1). For *Salmonella* Napoli–infected patients with recent travel histories, those who traveled within Italy were more likely infected with this serovar (adjusted odds ratio 7.82, 95% CI 1.00–61.25; p = 0.050) than patients who traveled abroad. Although patients infected with *Salmonella* Napoli were hospitalized at rates similar to those infected with other serovars (Table 1), they had a greater risk for development of bacteremia (adjusted odds ratio 4.25, 95% CI 3.07–5.88; p<0.001).

**Table 1:** Characteristics of patients infected with *Salmonella enterica* serovar Napoli and patients infected with the 5 most common other nontyphoidal serovars, Italy, 2011–2015*

Characteristic	Serovar Napoli, no. (%)	Other serovars, no. (%)	Total	p value	aOR (95% CI)
Sex					
M	525 (5.2)	7,470 (73.8)	10,119	0.001	1.17 (1.02–1.35)
F	383 (4.3)	6,521 (73.5)	8,876		1
Unknown	111 (4.5)	1,757 (70.5)	2,491		0.94 (0.75–1.18)
Age, y					
<1	69 (8.8)	491 (62.6)	784	<0.001	3.05 (2.22–4.18)
1–14	584 (5.6)	8,378 (80.2)	10,442		1.57 (1.28–1.92)
15–64	127 (3.1)	2,667 (65.6)	4,063		1
>65	195 (5.0)	2,447 (63.3)	3,863		1.76 (1.39–2.22)
Unknown	44 (1.9)	1,765 (75.6)	2,334		0.65 (0.45–0.92)
Geographic area					
North	893 (5.1)	12,708 (73.0)	17,397	<0.001	1.77 (1.43–2.20)
Central	102 (3.0)	2,540 (74.1)	3,428		1
South	24 (3.6)	500 (86.4)	661		1.63 (1.02–2.61)
Time of sampling					
Jan–Mar	12 (0.3)	3,623 (78.3)	4,626	<0.001	1
Apr–Jun	203 (4.4)	3,356 (73.2)	4,584		17.98 (10.02–32.26)
Jul–Sep	594 (8.8)	4,622 (68.5)	6,752		38.00 (21.41–67.44)
Oct–Dec	210 (3.8)	4,147 (75.1)	5,524		15.15 (8.45–27.17)
Period of sampling					
2011–2013	643 (4.3)	11,152 (73.9)	15,081	<0.001	1
2014–2015	376 (5.9)	4,596 (71.8)	6,405		1.4 (1.22–1.61)
Hospitalization					
Yes	368 (4.9)	5,453 (72.4)	7,533	0.583	ND
No	358 (4.9)	5,533 (75.1)	7,367		ND
Unknown	293 (4.4)	4,762 (72.3)	6,586		ND
Bacteremia					
Yes	962 (4.6)	15,527 (74.1)	20,962	<0.001	ND
No	57 (10.9)	221 (42.2)	524		ND
Unknown	6 (4.9)	75 (61.0)	123		ND
Total	1,019 (4.7)	15,748 (73.3)	21,486	ND	ND


*The 5 most common other serovars were the monophasic variant of Typhimurium, Typhimurium, Derby, Enteritidis, and Infantis. Hospitalization and bacteremia were not included in evaluation of risk factors because they were considered a consequence of the infection, rather than risk factors. aOR, adjusted odds ratio; ND, not determined.

Among nonhuman isolates, 15,579 *Salmonella* spp. were reported (7,563 animal, 5,496 food, 2,078 environmental, and 442 feed). These isolates included 187 *Salmonella* Napoli (89 animal, 76 environmental, 21 food, and 1 feed).

*Salmonella* Napoli ranked fifth (3.7%) among environmental *Salmonella* spp. and showed a significant increase from 1.6% in 2011 to 2.9% in 2015 (p<0.005). A total of 85% of Napoli isolates were from surface water. Multivariate analysis (Table 2) confirmed that Napoli strains were more commonly isolated during April–September (p<0.001) and less commonly in northern and southern Italy than in central Italy.

**Table 2:** Characteristics of environmental samples contaminated with *Salmonella enterica* serovar Napoli and samples with the 5 most common other nontyphoidal serovars, Italy 2011–2015*

Characteristic	Serovar Napoli, no. (%)	Other serovars, no. (%)	Total	p value	aOR (95% CI)
Geographic area					
Northern	37 (2.5)	531 (35.6)	1,493	<0.001	0.13 (0.06–0.26)
Central	16 (11.2)	38 (26.6)	143		1
Southern	23 (5.2)	151 (34.2)	442	0.001	0.25 (0.11–0.57)
Time of sampling					
Jan–Mar	4 (1.0)	200 (49.8)	402		1
Apr–Jun	28 (4.9)	169 (29.8)	567	<0.001	9.45 (3.15–28.35)
Jul–Sep	30 (4.7)	153 (24.2)	632	<0.001	10.8 (3.62–32.25)
Oct–Dec	14 (2.9)	198 (41.5)	477	<0.05	3.83 (1.22–12.06)
Period of sampling					
2011–2013	43 (3.6)	411 (34.5)	1,193		1
2014–2015	33 (3.7)	309 (34.9)	885	0.066	0.59 (0.33–1.04)
Total	76 (100.0)	720 (100.0)	2,078	ND	ND

*The 5 most common other serovars were the monophasic variant of Typhimurium, Typhimurium, Derby, Enteritidis, and Infantis. aOR, adjusted odds ratio; ND, not determined.

Among *Salmonella* spp. animal isolates, the frequency of *Salmonella* Napoli increased from 0.7% in 2011 to 1.3% in 2015. A total of 50% of Napoli strains were obtained in the wild, most (70%) from wild boars in northern Italy. The low proportion (0.4%) from food remained stable during 2011–2015. Of 21 Napoli samples, 7 were from shellfish. One alfalfa sample contaminated with Napoli was reported in southern Italy in 2014.

We performed pulsed-field gel electrophoresis for 182 *Salmonella* Napoli strains isolated from different sources. We detected 121 unique profiles; 32 identical profiles contained 2–13 isolates. Despite high genetic variability among strains, we identified 4 main clusters that had genetic homologies >80% and matched the 3 areas in Italy: cluster A in northern Italy, cluster B in central Italy, and clusters C and D in southern Italy (p<0.0001) (Figure). In southern Italy, all human and environmental isolates from northern Italy belonged to cluster A, and most in central Italy belonged to cluster B (p<0.0001). Most human strains isolated in southern Italy belonged to clusters A or D, and 90% of environmental strains belonged to cluster C (p<0.0001). The 4 isolates from vegetables in southern Italy belonged to clusters A, C, and D. Seven profiles with genetic homologies <80% were obtained from different sources in northern and southern Italy.

**Figure:**
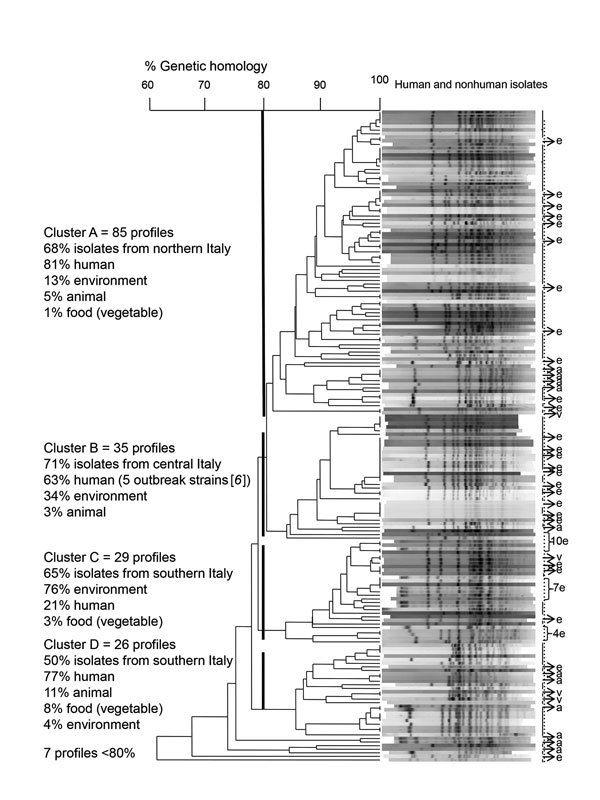
Dendrogram of 182 pulsed-field gel electrophoresis–based profiles of *Salmonella enterica* serovar Napoli strains isolated from human, environmental, animal, and food samples in Italy, 2011–2015. Four main clusters matched with the 3 main geographic areas in Italy (cluster A in northern Italy, cluster B in central Italy, and clusters C and D in southern Italy). Genetic analysis was based on 80% homology. Human strains (n = 124) are indicated by a solid vertical line. e indicates environmental strains (n = 46), a indicates animal strains (n = 8), and v indicates food (vegetable) strains (n = 4).

## Conclusions

Outbreaks of *Salmonella* Napoli infection reemerged in Italy during 2011–2015. In Italy, *Salmonella* Napoli patients accounted for 5.9% of salmonellosis cases reported during 2014–2015. France had highest percentage of infections (1.2%) during this period (http://atlas.ecdc.europa.eu/public/index.aspx). Consistent with a study conducted in Italy (*9*), we found that surface water, including irrigative water, rather than animal food, was the main cause of infection with *Salmonella* Napoli. On the basis of an outbreak in Sweden (http://www.epinorth.org/eway/default.aspx?pid_230&trg_Area_5328&MainArea_5260_5328:0:&4148_5326:58&Area_5328_5273:46553::0:5326:574:::0:0), reports of the Rapid Alert System for Food and Feed on vegetables from Italy contaminated with *Salmonella* Napoli, and that the fact that ≈30% of irrigative surface water in Italy is used for vegetables (https://www.istat.it/it/files/2014/11/Utilizzo_risorsa_idrica.pdf), we believe that ready-to-eat vegetables irrigated with contaminated surface water are the main food vehicle for *Salmonella* Napoli.

Marked seasonality of cases matching environmental reports might be caused by increased irrigation and consumption of ready-to-eat vegetables during summer and fall, and more frequent human contact with contaminated surface water during summer and fall. We also reported frequent isolation of *Salmonella* Napoli from wild boars, which suggests a role for dissemination through the environment (*12*). Human and environmental strains of *Salmonella* Napoli belonging mainly to cluster A in northern Italy and cluster B in central Italy suggest direct infection. Different clusters for human and environmental strains from southern Italy might indicate wider spread in the environment.

Heterogeneous sampling of nonhuman isolates through Italy was a limitation of this study. Travel history within Italy, rather than abroad, of patients infected with *Salmonella* Napoli increases public health concern in Italy. Uncontrolled spread of *Salmonella* Napoli through the environment and associated foodborne and waterborne outbreaks, the increasing proportion of young patients, and high risks for development of bacteremia make infections with *Salmonella* Napoli a serious public health concern in Italy.
